# Characteristics of Marine Gravity Anomaly Reference Maps and Accuracy Analysis of Gravity Matching-Aided Navigation

**DOI:** 10.3390/s17081851

**Published:** 2017-08-10

**Authors:** Hubiao Wang, Lin Wu, Hua Chai, Yaofei Xiao, Houtse Hsu, Yong Wang

**Affiliations:** 1 State Key Laboratory of Geodesy and Earth’s Dynamics, Institute of Geodesy and Geophysics, Chinese Academy of Sciences, Wuhan 430077, China; wanghb@whigg.ac.cn (H.W.); wulin1108@gmail.com (L.W.); hchai@whigg.ac.cn (H.C.); xiaoyaofei15@mails.ucas.ac.cn (Y.X.); hsuh@whigg.ac.cn (H.H.); 2 University of Chinese Academy of Sciences, Beijing 100049, China

**Keywords:** underwater navigation, gravity matching, marine gravity anomaly reference map, characteristic parameter, regional location accuracy evaluation

## Abstract

The variation of a marine gravity anomaly reference map is one of the important factors that affect the location accuracy of INS/Gravity integrated navigation systems in underwater navigation. In this study, based on marine gravity anomaly reference maps, new characteristic parameters of the gravity anomaly were constructed. Those characteristic values were calculated for 13 zones (105°–145° E, 0°–40° N) in the Western Pacific area, and simulation experiments of gravity matching-aided navigation were run. The influence of gravity variations on the accuracy of gravity matching-aided navigation was analyzed, and location accuracy of gravity matching in different zones was determined. Studies indicate that the new parameters may better characterize the marine gravity anomaly. Given the precision of current gravimeters and the resolution and accuracy of reference maps, the location accuracy of gravity matching in China’s Western Pacific area is ~1.0–4.0 nautical miles (n miles). In particular, accuracy in regions around the South China Sea and Sulu Sea was the highest, better than 1.5 n miles. The gravity characteristic parameters identified herein and characteristic values calculated in various zones provide a reference for the selection of navigation area and planning of sailing routes under conditions requiring certain navigational accuracy.

## 1. Introduction

The inertial navigation system (INS) is one of the major approaches for underwater navigation, but errors of the INS accumulate over time and increase gradually. The INS alone can hardly provide accurate navigation information over a long period of time. To compensate this deficiency of the INS, various integrated navigation systems centered on inertial navigation have been developed, and an inertial/gravity integrated navigation system based on characteristics of the geophysical field has become an effective approach for navigation by underwater submarines under conditions of long-range underwater navigation over a long period [1,2,3,4,5,6,7,8]. The geophysical field-aided navigation method primarily uses gravity and magnetic fields and sea floor terrain data measured by an underwater submarine to perform matching and comparison with graphical data stored in advance, thus obtaining accurate estimates of locations and correcting the INS errors. Such methods are mainly based on sufficiently numerous variations of measured physical or geometrical parameters in spatial distribution, and, through comparison with pre-stored reference maps, correct matching is achieved. The marine gravity field reference map is a tool that describes the spatial distribution of that field, and possesses unique advantages such as passivity, stability, anti-interference, and irregularity of the distribution when using a gravimeter to acquire gravity information at the location of an underwater submarine. As a result, gravity field matching-aided inertial navigation has gradually become one of the preferred methods to improve the location accuracy of underwater submarines [9,10].

Gravity matching-aided inertial navigation is a technique that uses characteristics of the earth’s gravity field to acquire location information of the carrier. The gravity reference map is the basis for performing such navigation and is one of the four primary factors (inertial navigation, gravimeter, reference map, and matching algorithm) of the marine gravity-aided INS. The gravity reference map includes parameter data of the gravity field such as the deflection of the vertical, gravity anomaly, and gravity gradient. The resolution and accuracy of such data are closely related to the location determination success rate and accuracy of matching-aided navigation. At present, the marine gravity anomaly reference map is one of the most important background fields in geophysical-aided navigation [11,12,13].

The matching algorithm and precision of the gravimeter and INS used are important in the matching performance. However, the gravity anomaly reference map and characteristics of the matching zone also have a major influence on the location determination success rate and accuracy of the gravity anomaly-aided navigation system. When navigation sensors (gravimeter or INS) have the same accuracy, the gravity anomaly reference map with its variable accuracy and resolutions and sailing regions with different gravity anomaly characteristics may produce different matching-aided navigation effects. Characteristics of the gravity anomaly are used to perform underwater-aided inertial navigation, and various national and international experts and scholars have explored this topic continuously and made extraordinary achievements. These include studies on the applicability of a gravity matching algorithm for INS with various accuracies [14], selection methods of matching zones based on characteristic analysis of the gravity reference map [15,16,17], and the influence of gravimeter measurement error on matching performance [18]. In research regarding the influence of the gravity anomaly characteristics on the location accuracy of gravity matching, some scholars have produced qualitative results, i.e., the greater the gravity variation, the better the location accuracy of gravity matching [19]. However, using a navigation sensor and gravity anomaly reference map under current technical conditions, how to quantitatively evaluate the location accuracy of gravity matching in different marine gravity anomaly characteristic zones remains a problem in need of solution.

We used marine gravity anomaly data provided by the Scripps Institute of Oceanography (USA) and studied the influence of these data on the gravity-aided INS from the perspective of the gravity anomaly reference map. Based on newly constructed gravity anomaly characteristic parameters, using an example of 13 zones in the China Western Pacific area (105°–145° E, 0°–40° N), those characteristics were calculated for the zones. We deduced the theoretical and actual location accuracy that gravity matching-aided navigation could attain in the zones under current conditions, and the effects of the characteristic parameters on gravity matching were validated via gravity matching simulation. Thus, the feasibility and performance of gravity-aided navigation in different navigation regions were evaluated in advance by referring to the location accuracy of gravity matching in the zones. This provides a reference for planning navigation regions and designing navigation routes for underwater submarines.

## 2. Principles of Gravity-Aided Navigation and Characteristic Analysis Method of a Marine Gravity Reference Map

### 2.1. Principles of Gravity-Aided Navigation

**Figure 1:**
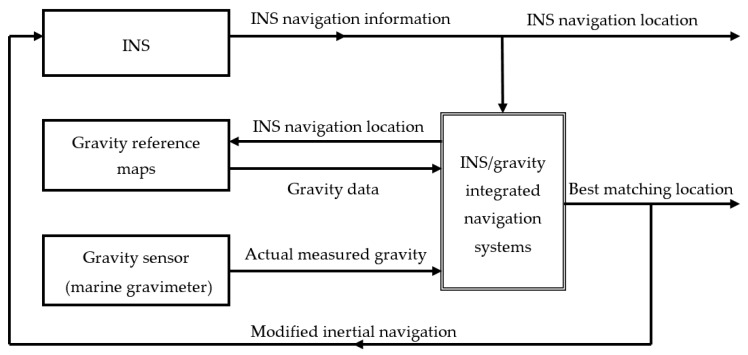
The basic principle of gravity matching-aided navigation.

Gravity anomaly-aided INS is a navigation technique that uses a high-resolution gravity anomaly reference map and acquires location information of the carrier based on information database characteristics of the gravity anomaly reference map. Gravity anomaly matching-aided navigation uses various information processing methods to compare actually measured marine gravity anomaly information with gravity anomaly information stored in the marine gravity anomaly reference map. The degree of fit between the two is determined according to certain principles, and the best matching point is determined. Figure 1 illustrates the basic principle of the gravity anomaly-aided matching navigation.

The design of the gravity anomaly matching algorithm relates to techniques such as mathematical descriptions and extraction of marine gravity characteristics, error analysis of gravity sensor measurement, best matching principles, and criteria of effective location determination for preventing false locations. Applicable methods include correlation analysis, multi-model self-adaptive Kalman filter estimation, neural networks, and statistical pattern recognition [20,21,22,23,24,25]. The basic principles of these methods are all based on the following gravity anomaly matching method:

(1) $$Y=G(x_{t},y_{t})-G_{M}(x_{i},y_{i})$$

where $G(x_{t},y_{t})$ is the gravity anomaly measured at the actual location $\left(x_{t},y_{t}\right)$ of the carrier, and $G_{M}(x_{i},y_{i})$ is the gravity anomaly read from a reference map according to the location $\left(x_{i},y_{i}\right)$ indicated by the INS. Based on the sequence of Y values, correlation processing or Kalman filter algorithms are frequently used to perform matching-aided navigation.

Figure 1 and Equation (1) illustrate the basic principle of the INS/gravity integrated navigation system, but the location reference is at the sea surface level. The submarine sails in different dive depths, so the submarine’s trajectory in the ocean is located in a 3D space. Gravity anomaly observations at the underwater submarine depth and the gravity anomaly reference map at sea level are located on different datums; thus, the observed gravity at the submarine location is less than the value observed on the sea’s surface due to the gravity of a layer of seawater above the submarine. Submarine dive depth is generally between 150 m to 500 m. The depth is computed from the direct measurements of ambient sea water pressure via standard equations for the properties of sea water, and the error is usually less than 0.1% [26]. In the actual case, the gravity anomaly value measured at the submarine’s location (which is at a certain underwater depth) and the marine gravity anomaly reference map (which is located at sea level) have to be converted to the same level to be used. According to the submarine depth meter measurement and other related information, using the gravity anomaly upward extension method, we converted gravity anomaly observations to the corresponding sea level with the gravity anomaly reference map, thus converting the submarine’s location from 3D space to 2D sea level. Usually, an upward extension (such as Poisson integral method) is used. The gravity anomaly at submarine dive depth is converted to that at the sea surface by upward extension; the gravity anomaly error caused by upward extension from a 500 m depth is less than 0.5 mGal [27].

There are many factors that affect the location accuracy of an INS/gravity integrated navigation system, such as the accuracy of gravimeter observation, INS accuracy, and resolution and accuracy of the gravity anomaly reference map. Different matching algorithms also have some influence on location accuracy. When sailing over a long distance and a long period of time, the drift of gravimeter is also an important factor that can affect the location accuracy of gravity matching. Usually, monthly drift of the gravimeter is less than 3 mGal, and have good linear technical characteristics. If the submarine sails long distances underwater, such as sailing for 2 months, the gravity drift can be up to 6 mGal; but taking into account the almost linearity and variation regularity of the zero drift, the drift of the gravimeter can be treated and controlled within ~1–2 mGal [28,29].

Relative to the marine gravimeter observation accuracy of 1–2 mGal and gravity reference map accuracy of 3–8 mGal, the gravity anomaly error caused by upward extension and the marine gravimeter drift error are relatively small and can be integrated in the marine gravimeter observation error or gravity reference map error. Using the INS/gravity integrated navigation system, two-dimensional coordinates (longitude and latitude) based on the sea surface are given; combined with the depth meter on the submarine, the 3D location of the submarine is determined.

No matter which method is adopted, the core concept of the gravity anomaly matching algorithm is based on the fact that the gravity anomaly in navigation regions of the underwater submarine has various characteristics, and different characteristics alter gravity matching navigation accuracy. Thus, it is necessary to construct a proper parameter that evaluates characteristics of the gravity anomaly zone and analyzes the influence of characteristic parameters on the location accuracy of gravity matching [30,31].

### 2.2. Characteristic Analysis of Marine Gravity Reference Map

Evaluation of the gravity anomaly reference map characteristics may be described from various aspects such as statistical, correlation, frequency, and fractal. Often, when referring to the definition of the topographic characteristic parameter, parameters such as standard deviation, roughness, information entropy, correlation coefficient, and topographic slope are largely used to describe the information characteristics of a zone. These characteristic parameters represent the discreteness of the data in the matching zone, the similarity between different data, and the magnitude of change [32]. The gravity anomaly-aided navigation reference map is often stored using a grid matrix method. To analyze statistical features of the local gravity anomaly, the size of the local calculation window is defined as a *m × n* grid. One characteristic parameter calculated via various elements of the calculation window is used to represent the parameter value of the window zone. By moving the calculation window across the grid points of the entire selected matching zone, each statistical parameter of the gravity field in that zone is calculated. The calculation method of standard deviation and absolute roughness among the characteristic parameters of gravity anomaly Δg is shown below, and new characteristic parameters are constructed based thereon.

(a) Standard deviation of gravity anomaly.

(2) σ=(1mn−1∑i=1m∑j=1n(Δg(i,j)−Δg¯)2)12,Δg¯=1mn∑i=1m∑j=1nΔg(i,j)

Here, Δg(i,j) is the gravity anomaly at coordinates (i,j) of the grid point in the local calculation window, where $\bar{\Delta g}$ is the average gravity anomaly in that window and σ is standard deviation of the gravity anomaly in the window. σ primarily reflects the dispersion and variation of an entire zone in part of the data map.

(b) Absolute roughness of gravity anomaly.

(3) $$r_{\lambda }=\frac{1}{(m-1)n}\sum_{i=1}^{m-1}\sum_{j=1}^{n}\left|\Delta g(i,j)-\Delta g(i+1,j)\right|$$

(4) $$r_{\varphi }=\frac{1}{m(n-1)}\sum_{i=1}^{m}\sum_{j=1}^{n-1}\left|\Delta g(i,j)-\Delta g(i,j+1)\right|$$

(5) $$r=(r_{\lambda }+r_{\varphi })/2$$

Here, r is absolute roughness of the gravity anomaly in the local zone, and $r_{\lambda }$ and $r_{\varphi }$ are the absolute roughnesses of that anomaly along longitudinal and latitudinal directions, respectively. r reflects average smoothing of the gravity anomaly within the calculation window, which can describe relatively subtle local variations.

(c) Integration of gravity anomaly standard deviation and absolute roughness.

The standard deviation of gravity anomaly is a measure of deviation between each data and the average in one zone, and represents overall characteristics of this zone at macroscopic scale. Absolute roughness of the gravity anomaly measures variation between adjacent observation points in the zone, and represents the magnitude of variation in one zone at microscopic scale. In gravity matching-aided navigation, actual resolution of the marine gravity anomaly reference map is typically 1′ × 1′ or 2′ × 2′ (1′ = 1 n mile = 1853 m). Though we may use mathematical interpolation to make the resolution reach 0.1′ × 0.1′ or even higher, this resolution is not real and cannot increase the actual location accuracy. In gravity matching-aided navigation, maximum accuracy of a half grid may theoretically be reached. However, if the gravity anomaly variation between adjacent grid points is very small or even fails to attain the variation of measurement accuracy for the gravimeter at 1 mGal, the variation of gravity anomaly between adjacent grid points may be overwhelmed by measurement noise of the gravimeter. In such a case, when the underwater submarine moves the distance of one grid, the gravity matching-aided navigation system cannot effectively distinguish this difference of gravity variation to accurately determine the location. Therefore, in that system, gravity anomaly characteristics must be evaluated at macroscopic scale, and the characteristics of adjacent grids must be considered at microscopic scale.

**Figure 2:**
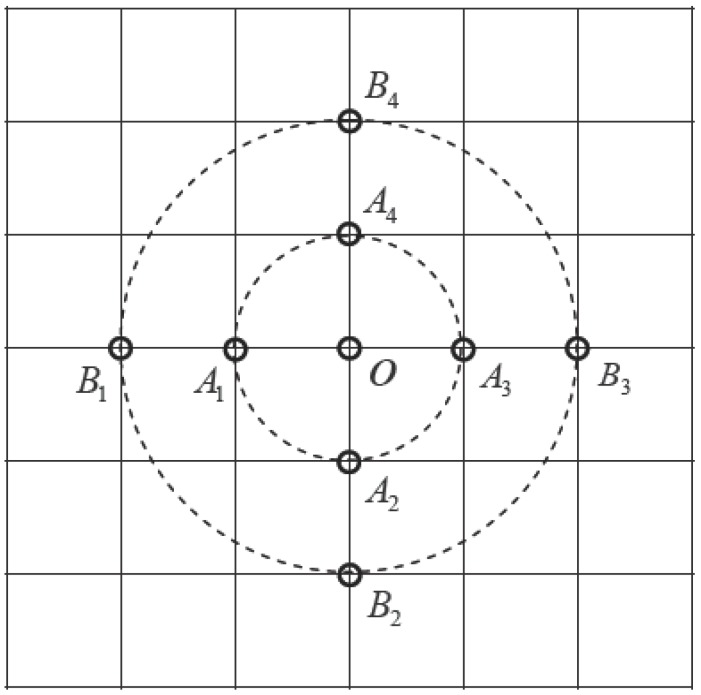
Principal schematic view of new characteristic parameters.

Based on the aforementioned reasons, and considering that the resolution of the marine gravity anomaly reference map (grav.img.24.1) used is 1′ × 1′, the gravity anomaly characteristic parameter calculation shown by Figure 2 was formulated. The grid interval in the figure is 1′. When the underwater submarine moves to point O in the figure, the opportunity to move in any direction in the next step is equal. For example, if one grid interval is used as reference, the deviation of the gravity anomaly between O and points $A_{1}$, $A_{2}$, $A_{3}$, and $A_{4}$ are determined. If two grid intervals are used as reference, the deviation of the gravity anomaly between O and points $B_{1}$, $B_{2}$, $B_{3}$, and $B_{4}$ are determined. Similarly, the characteristics of adjacent grids can be determined, thereby providing a reference for the location accuracy of the underwater submarine. Assuming that the gravity anomaly of O is Δg(i,j), (i,j) represents the coordinates of O. Here, *k* grid intervals are used as an example, and the following gravity anomaly characteristic parameter Γ is designed, which characterizes the difference of gravity anomaly between a certain point and a nearby measurement point within a certain distance:

(6) $$S_{\lambda }={\left[\Delta g(i,j)-\Delta g(i-k,j)\right]}^{2}+{\left[\Delta g(i,j)-\Delta g(i+k,j)\right]}^{2}$$

(7) $$S_{\varphi }={\left[\Delta g(i,j)-\Delta g(i,j-k)\right]}^{2}+{\left[\Delta g(i,j)-\Delta g(i,j+k)\right]}^{2}$$

(8) $$\Gamma (i,j)=\sqrt{(S_{\lambda }+S_{\varphi })/4}$$

$S_{\lambda }$ is the sum of the difference of two squares of the gravity anomaly in the longitudinal direction, $S_{\varphi }$ is the same but in the latitudinal direction, and Γ(i,j) is the gravity anomaly characteristic parameter for point O(i,j). Equation (8) only gives the characteristic parameter based on a single observation point. To evaluate overall characteristics of a relatively large local area in an *m × n* grid using this characteristic parameter, the following equation was constructed:

(9) Γk=(1mn−1∑i=1m∑j=1nΓ2(i,j))12

## 3. Characteristics of the Marine Gravity Reference Map and the Location Accuracy of Gravity Matching

### 3.1. Marine Gravity Reference Map

**Figure 3:**
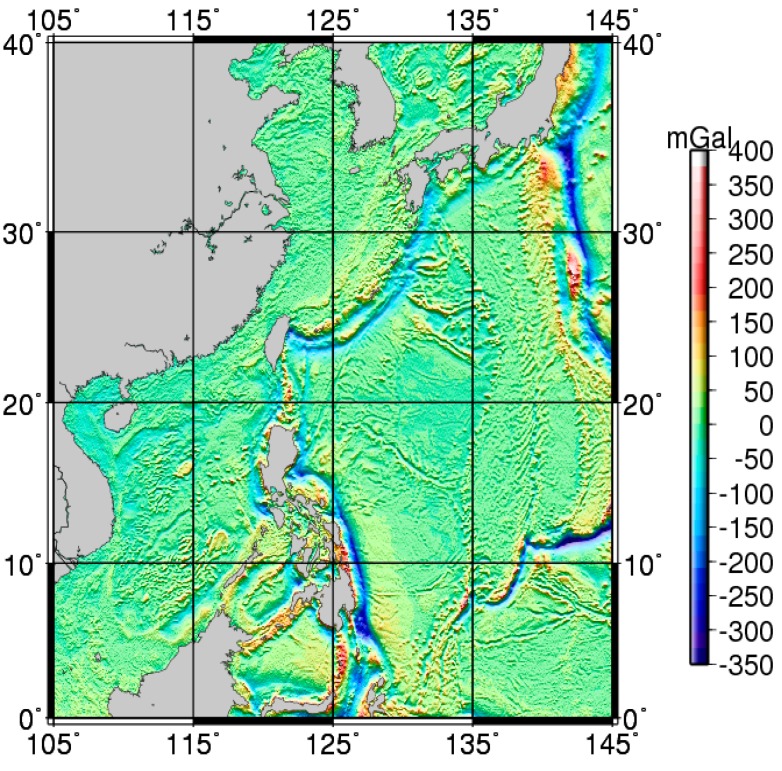
Schematic view of gravity anomaly reference map.

Underwater gravity matching-aided INS is a systematic project, and our research develops with the gravity sensor system, inertial sensor system, and global marine gravity field measurements. As the techniques of satellite altimetry and satellite gravity have developed, the resolution of the marine gravity field has reached 2′ × 2′ and, based on data processing and further refinements, the Scripps Institution of Oceanography and University of California—San Diego proposed the 1′ × 1′ global marine gravity anomaly model grav.img.24.1 [33], whose accuracy reaches 3–8 mGal [34,35,36]. Figure 3 is a schematic view of the gravity anomaly reference map in the China Western Pacific area. Currently, measurement accuracy of the latest LaCoste & Romberg-type and KSS31-type marine gravimeter reaches ±1.0 mGal under relatively poor sea conditions. Take into consideration the influences of individual errors (zero drift correction, Eotvos correction, inaccurate location determination, and spatial correction) in marine measurement, an accuracy of ±1.0 mGal–±2.0 mGal may be attained [37]. These allow for the use of the gravimeter and marine gravity reference map in underwater-aided navigation.

### 3.2. Characteristic Value of the Marine Gravity Reference Map

**Figure 4:**
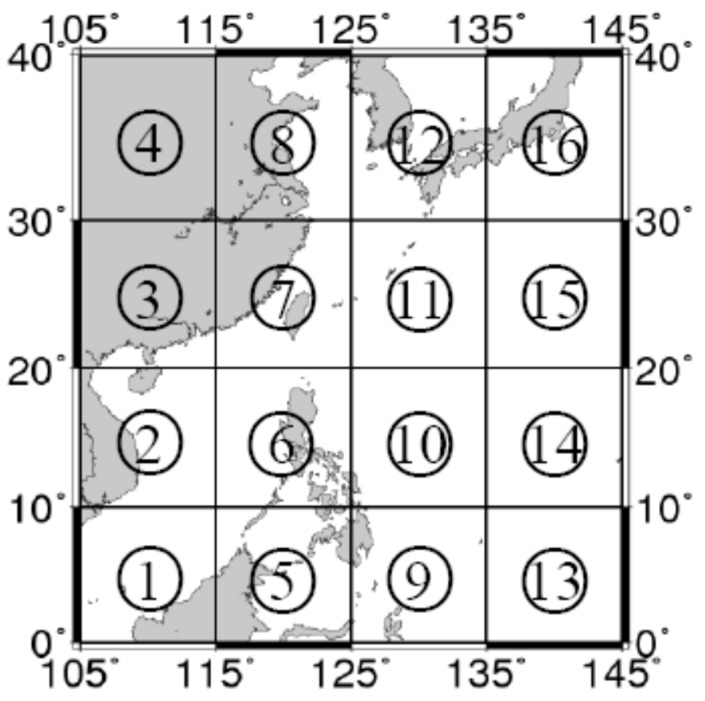
Schematic view of zones in the China Western Pacific area.

Many scholars have used various characteristic parameters to qualitatively illustrate the relationship between the characteristics of the marine gravity anomaly reference map and the location accuracy of gravity anomaly matching. However, many parameters are able to describe characteristics of the gravity anomaly reference map, and different parameters may characterize properties with variable emphases. To analyze correlation between the characteristic parameter developed herein and the location accuracy of the gravity anomaly matching, and to analyze the performance of that matching in the China Western Pacific area (105°–145° E, 0°–40° N), that area was divided into 16 regions of size 10° × 10° (Figure 4). Among these, regions ③, ④, and ⑧ were not studied because most of their areas consisted of land. Gravity anomaly characteristics of the other 13 regions were determined and analyzed. Quantitative analysis of the accuracy of gravity anomaly matching-aided navigation in various regions was done based on validation from the simulation.

Equations (2) and (9) were used to solve characteristic parameters σ and Γ for the aforementioned 13 regions, where $\Gamma_{1}$ corresponds to *k* = 1 in Equations (6)–(9). This gives variance characteristics between point O in Figure 2 (used as the center) and nearby points *A_1_–A_4_* in one grid interval. Similarly, $\Gamma_{2}$, $\Gamma_{3}$, $\Gamma_{4}$, $\Gamma_{5}$, and $\Gamma_{6}$ were obtained. Specific results are listed in Table 1.

From Table 1, the characteristic parameter Γ($\Gamma_{1}$ through $\Gamma_{6}$) in 13 regions were included, and corresponding characteristic values all increased gradually from $\Gamma_{1}$ to $\Gamma_{6}$ in the same region. This indicates that characteristics of the gravity anomaly became more important as the interval increased from 1′ to 6′. Overall, values of Γ in regions ⑤, ⑥, ⑨, and ⑯ were the largest, and those in regions ① and ⑩ the smallest. This was relatively consistent with characteristic parameter σ, but there was a difference. For example, values of σ in regions ①, ②, and ⑫ were the smallest, all <30.0. However, regarding Γ, using $\Gamma_{4}$ as an example, its values in regions ①, ②, and ⑩ were the smallest, all <7.8.

### 3.3. Location Accuracy of Gravity Matching Based on Simulation Analysis

To illustrate the quantitative relationship between characteristic parameter and the location accuracy of gravity matching, we selected region ⑤ with the most important characteristic and region ⑩ with the least important. Further, one route each was selected for simulation analysis, and the simulation validation of gravity anomaly matching was done based on the SITAN algorithm of the Kalman filter principle [38,39,40,41].

The speed of submarines underwater is usually around 10.0 knots (1 knot = 1 n miles/h), but in long-range underwater navigation for a long period time, such as sailing for 1 month, where the submarine sometimes stops, the average speed may be about 2.5 knots. After long-range underwater navigation for a long period time, this matching location error, which is passed to the INS for correction, may reach 1–2 n miles. The effect of gravity matching in the case of a relatively large INS initial location error (up to 1.0 n mile) is tested. In the parallel Kalman filter matching algorithm, system noise and observation noise belong to priori knowledge, mainly based on subjective estimates of observations; these refer to the accuracies of the marine gravimeter and marine gravity reference map, and are empirically determined. Based on the above considerations, we set the following initial conditions for the simulation: the underwater submarine moves at constant speed 2.5 n mile/h under instruction of the INS, the INS initial location error is 1.0 n mile, location accuracy is 0.1 n mile/h, system noise is 1.0 mGal^2^, and observation noise is 10.0 mGal^2^.

Here, the observation noise includes error of the gravity background field database, interpolation error, and measurement error of the gravimeter. Assuming 50% CEP (circular error probable) of the inertial navigation is 15 n mile, 99% of the CEP is 38 n miles. The simulated observation value of the gravity anomaly is obtained based on the bilinear interpolation value for the simulated actual location in the corresponding gravity anomaly reference map and observation noise of the gravity anomaly. For the same reference map under different sampling distances, matching performance of the data may vary. Corresponding to the gravity field background database, one observation value is established for every ~1 n mile of submarine travel. After the initial location and sailing direction are given, the SITAN algorithm is used to simulate and calculate the location of inertial navigation at each moment.

**Figure 5:**
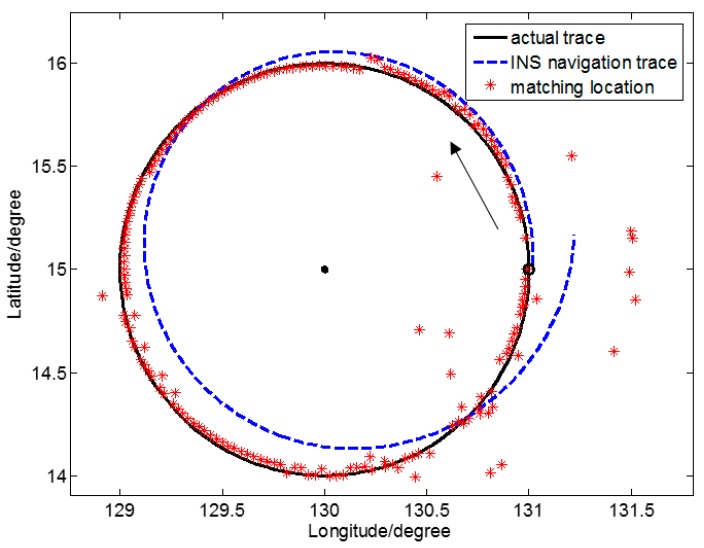
Simulated trace of closed sailing route gravity matching (sailing route A).

**Figure 6:**
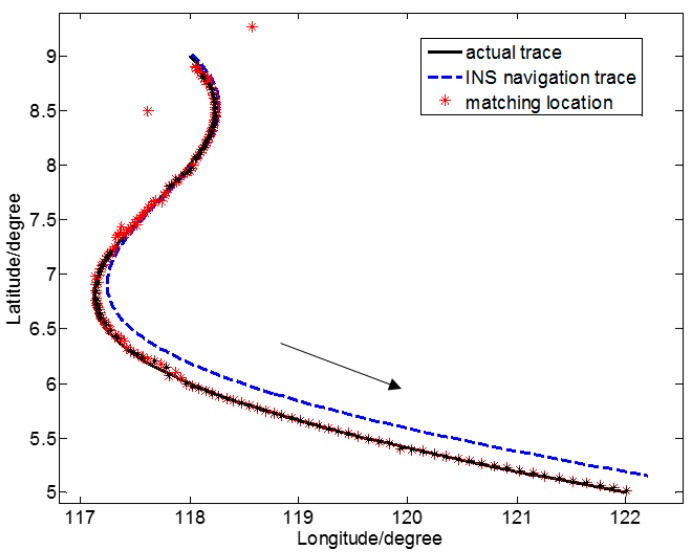
Simulation trace of irregular sailing route gravity matching (sailing route B).

Figure 5 is a schematic view of the location determination results of gravity anomaly matching for one closed simulation sailing route in region ⑩, where the gravity anomaly characteristic is the least important. In particular, • is the center of a circle, ○ is the starting point of the sailing track, and → is the direction of motion of the underwater submarine. Figure 6 is a schematic view of the location determination results of gravity anomaly matching for one irregular curved simulated sailing route in region ⑤, where the gravity anomaly characteristic is the most important. Here, – – represents the simulated actual trace, - - - is the simulated INS navigation trace, and * are the locations determined by gravity anomaly matching.

The SITAN algorithm is based on the Kalman filter algorithm. The Kalman filter is relatively loose in defining the initial value. However, because of unpredictability of the system, an inaccurate initial value from the Kalman filter is of little influence. Because the error between the estimate of initial status $X_{0}$ and that of initial variance $P_{0}$ in the Kalman filter equation is relatively large, the difference between the initial value of the Kalman filter estimate and the actual initial value is relatively large. As the number of iterations increases, $P_{0}$ may decrease gradually, and prediction accuracy may increase continuously. However, this improvement only shows apparent reactions in the first few steps, and after several iterations, the filter gain stabilizes, and the error may rapidly converge to a certain domain. Such initial value configuration may only affect the filter the first few times, and often the tracking goal may be well predicted after several iterations. In the simulated sailing routes of Figure 5 and Figure 6, the initial location determination errors are relatively large. These, however, all converge rapidly, and the target location is tracked.

Considering the relatively large initial matching error of the Kalman filter, and to more reasonably assess the accuracy of location determination of the two sailing routes, we calculated statistical accuracy starting with the matching location where effective location determination was achieved for the first time. Table 2 gives statistical results of location accuracy of gravity anomaly matching for the two sailing routes in two representative characteristic regions.

## 4. Discussion

In Table 2, the measurement point serial number of the first effective matching along sailing route A is 12, and that number along sailing route B is 8. This indicates that along route B, where the characteristic was more important, location determination by matching converged more quickly.

At present, observation accuracy $\sigma_{1}$ of the gravimeter is ~1–2 mGal, and accuracy $\sigma_{2}$ of the marine gravity anomaly reference map is ~3–8 mGal. Further, accuracy $\sigma =\sqrt{\sigma_{1}{}^{2}+\sigma_{2}{}^{2}}$ after the two are integrated is ~3.1–8.3 mGal. Then, only when the actual gravity anomaly difference between two consecutive observation locations is not <σ, characteristics will not be masked by error noise of the gravimeter and gravity reference map. If the actual gravity anomaly difference is 1–2 mGal larger than σ, i.e., if it is >9.3 mGal, the characteristic may appear.

If $\Gamma_{k}\geq 9.3$, overall accuracy of the gravity anomaly matching navigation in the region may reach a location accuracy of $\frac{k}{2}$ grid (1 grid = 1′ = 1 n mile). In Table 1, for region ⑩ where sailing route A is located, $\Gamma_{6}=8.23<9.3$ (*k* = 6) and, referring to Table 2, the root mean square (RMS) of the location error is only 5.36 n mile, larger than $\frac{k}{2}$ grid. That is lower than the location accuracy of 3 n mile. In Table 1, for region ⑤ where sailing route B is located, $\Gamma_{3}=10.31>9.3$ (*k* = 3) and, referring to Table 2, the RMS of the location error is 1.51 n mile, an approximate location accuracy of $\frac{k}{2}$ grid.

Table 1 reveals that values of characteristic parameters $\Gamma_{4}$ through$\Gamma_{6}$ in regions ⑤, ⑥, ⑦, ⑨, ⑮, and ⑯ are all >9.3, indicating that these regions have relatively satisfactory effects of gravity anomaly matching navigation. Location accuracies of the gravity matching in regions ⑤ and ⑨ are the highest, and those in regions ① and ⑩ the lowest. From Table 1, the characteristic parameters σ and Γ have relatively strong correlation, and the larger the Γ at the measurement point of the navigation trace, the stronger the ability of the system to resist noise. Characteristic parameter σ may qualitatively represent the quality of navigation in each region, and parameter $\Gamma_{k}$ may quantitatively represent the location accuracy in each region. The location where data in bold first appears in Table 1 represents the potential location accuracy of the corresponding region. For example, the 11.07 in bold is located in $\Gamma_{5}$ for the first time in region ⑭, so the location accuracy in region ⑭ is 5/2 = 2.5 grids, which refers to 2.5′ and is ~2.5 n miles. For example, again in region ⑭, $\Gamma_{6}$ = 12.94, which is greater than the corresponding value of $\Gamma_{5}$ (i.e., 11.07). This indicates that the reliability of the location accuracy of 3 n miles in this region is greater than that of the location accuracy of 2.5 n miles. The greater the characteristic parameter of gravity anomaly in the region of Table 1, the greater the reliability of navigation.

According to the aforementioned analysis, with reference to the gravity anomaly characteristic parameters in Table 1 and based on the determination rule of $\Gamma_{k}\geq 9.3$, Table 3 gives the location accuracy of gravity anomaly matching in different parts of the China Western Pacific area. In this area, the location accuracy of regions ① and ⑩ was the lowest, >3.0 n miles.

Because the performance of location determination by matching is closely related to variation of the gravity anomaly in a region where the underwater submarine is located, the feasibility of navigation using gravity anomaly matching must be evaluated in advance for the navigation region. Given the existing gravimeter accuracy $\sigma_{1}$ (1–2 mGal) and marine gravity anomaly reference map (resolution 1′ × 1′, accuracy $\sigma_{2}$ 3–8 mGal), maximum theoretical location accuracy should be a half grid (0.5 n mile). The simulated location accuracy of most regions in the present work was within 1.0–4.0 n mile. The location accuracy of an actual marine experiment in a certain characteristic region was 2–3 n mile [42]. If actual gravimeter measurement is done in advance and a gravity anomaly reference map with resolution 1′ × 1′ is constructed, accuracy $\sigma =\sqrt{\sigma_{1}{}^{2}+\sigma_{2}{}^{2}}$ after integration of the gravimeter accuracy $\sigma_{1}$ and the gravity reference map accuracy $\sigma_{2}$ is ~1.4–2.8 mGal. Referring to Table 1, most regions in the China Western Pacific area satisfied the condition $\Gamma_{2}>3.8$, which yields a location accuracy of ~1 n mile.

In previous studies [14,15,16,17,18,19], the relevant factors influencing the location accuracy of gravity matching were studied from different angles, and these are good references for the study of the INS/gravity integrated navigation system. However, these studies have some limitations on the setting of gravity anomaly characteristic parameters and only give qualitative guidance, not quantitative results of the location accuracy for different regions. The characteristic parameters designed by this study consider the overall macroscopic factors, as well as the characteristics of adjacent grids at a microscopic scale. We also present the quantitative relationships between the characteristic value of gravity anomaly and the location accuracy of gravity matching-aided navigation. These results are improvements over those from previous studies.

## 5. Conclusions

Based on the designed characteristic parameter of gravity anomaly, zones of the China Western Pacific area were analyzed to give characteristics of the gravity anomaly across different regions. Matching navigation simulation was done for two sailing routes in different characteristic regions, and correlation between the characteristic value of gravity anomaly and matching error was determined. This shows that this characteristic parameter can provide overall location accuracy in different regions, thereby giving a reference for the selection of matching regions and planning of sailing routes with a certain navigation accuracy.

This paper presents a new characteristic parameter and examined the quantitative relationship between the characteristic value of gravity anomaly and the reliability and location accuracy of gravity anomaly matching-aided navigation. The optimal accuracy of that navigation depends on the minimum resolution of the gravity anomaly reference map and is closely related to variation of the gravity field in the region surrounding the sailing route of the underwater submarine. The marine gravity anomaly data from the Scripps Institute of Oceanography constitute one of the most effective global datasets. As satellite height measurement data are continuously supplemented and accumulated, and as new algorithms and models improve, accuracy and resolution of the marine gravity field may gradually improve, and the location accuracy of gravity matching-aided navigation based on gravity anomaly characteristics may also improve.

## Figures and Tables

**Table 1:** Characteristic parameters of gravity anomaly in various regions of the China Western Pacific area.

	①	②	⑤	⑥	⑦	⑨	⑩	⑪	⑫	⑬	⑭	⑮	⑯
σ	20.8	23.0	60.3	55.5	37.7	78.0	38.8	35.3	27.7	31.5	54.8	60.6	79.7
$\Gamma_{1}$	1.78	2.03	3.46	3.24	2.47	3.61	1.49	2.04	2.10	2.05	2.31	2.47	2.88
$\Gamma_{2}$	3.52	4.05	6.95	6.49	4.93	7.24	2.98	4.08	4.19	4.10	4.62	4.96	5.77
$\Gamma_{3}$	5.10	5.98	10.31	9.65	7.28	10.82	4.43	6.08	6.20	6.06	6.89	7.40	8.62
$\Gamma_{4}$	6.48	7.74	13.49	12.63	9.45	14.26	5.80	7.98	8.05	7.89	9.05	9.77	11.36
$\Gamma_{5}$	7.68	9.31	16.44	15.41	11.38	17.51	7.07	9.75	9.70	9.58	11.07	12.01	13.94
$\Gamma_{6}$	8.71	10.68	19.17	17.96	13.08	20.57	8.23	11.37	11.16	11.11	12.94	14.13	16.36

**Table 2:** Location accuracy of gravity matching for two sailing routes in different characteristic regions (unit: n mile).

Sailing Route	Starting Point	Mean	STD	RMS
A	12	2.59	4.69	5.36
B	6	1.23	0.86	1.51

**Table 3:** Location accuracy of gravity matching in various regions of China Western Pacific area (unit: n mile).

	①	②	⑤	⑥	⑦	⑨	⑩	⑪	⑫	⑬	⑭	⑮	⑯
**Accuracy**	>3.0	<2.5	<1.5	<1.5	<2.0	<1.5	>3.0	<2.5	<2.5	<2.5	<2.5	<2.0	<2.0
